# Importance of Developing New Tools to Assess Cardiac Amyloidosis Using Echocardiography

**DOI:** 10.1111/echo.70271

**Published:** 2025-08-25

**Authors:** Claudio Tinoco Mesquita, Andréa Rodrigues da Costa, Roberto Magalhães Saraiva

**Affiliations:** ^1^ Antonio Pedro University Hospital Fluminense Federal University Niterói Brazil; ^2^ Clinical Research Laboratory in Chagas Disease Evandro Chagas National Institute of Infectious Diseases Oswaldo Cruz Foundation Rio de Janeiro Brazil

1

Cardiac amyloidosis (CA) is one of the infiltrative cardiomyopathies and is caused by an abnormal accumulation of insoluble aggregates of misfolded proteins within the myocardium. Although considered a rare disorder, its prevalence has increased over the last decades due to increased detection, as a consequence of enhancement in imaging technologies, and population aging [1, 2]. Specifically, in the US population, there was an increase in the prevalence rate (8 to 17 per 100 000 person‐years) and incidence rate (18 to 55 per 100 000 person‐years) from 2000 to 2012 [3].  As CA is frequently undetected and portends a poor prognosis with a high mortality rate, early detection is of paramount importance in order to introduce therapies that can improve survival and quality of life [4]. Unfortunately, CA is still overlooked among patients with heart failure with preserved ejection fraction.

Echocardiography carries a Class 1B recommendation for diagnosing CA and is the typical first cardiac imaging modality for these patients [5]. In order to increase CA suspicion and diagnosis, several red flags on echocardiography were described by the The American Society of Echocardiography: increased left ventricular (LV) and right ventricular (RV) wall thickening, biatrial enlargement, increased LV wall thickness with low‐voltage criteria on associated electrocardiogram, diastolic dysfunction (≥grade II) with elevated LV filling pressures, severely reduced mitral annular tissue Doppler velocities, reduced global longitudinal strain (GLS) with apical sparing, low‐flow low‐gradient aortic stenosis, pericardial effusion, increased atrial septal thickness, diffuse valve thickening, and preserved ejection fraction with low stroke volume index [6]. However, the overlapping symptoms with other cardiac pathologies common in elderly population often result in diagnostic delays [2]. Therefore, new tools to facilitate CA suspicion during echocardiography are of great importance. The paper by Lin et al. [7] included 72 patients with primary light chain amyloidosis (pAL), with CA diagnosis confirmed by non‐cardiac tissue biopsy in 51 of them, and described a nomogram model with a very good diagnostic performance. After evaluating the univariate association with CA diagnosis of several echocardiographic parameters, the authors proposed a model which included relative wall thickness (RWT), the ratio of mitral peak flow velocity in early diastole (E) to the pulsed tissue Doppler early diastolic peak velocity (e’) at the interventricular septal mitral annulus (E /e’ sep), ejection fraction to peak GLS ratio (EFSR) and RV fractional area change. The study of the combined predictive value of several echocardiographic parameters in CA is novel and very important, as the predictive value for CA diagnosis of the proposed nomogram was superior to the one observed by single echocardiographic parameters. The model combines parameters that evaluate LV hypertrophy, RV systolic function, and LV diastolic and systolic function measured by speckle tracking echocardiography, all of them associated with CA. The EFSR was previously described as a sensitive marker of CA [8].

The authors must be congratulated for their important effort, but there are some limitations to the nomogram's accuracy. One point to be observed is the absence of left atrial (LA) strain in the proposed model. There is some evidence from recent studies, such as Liu et al. [9] and Aimo et al. [10], indicating that LA strain is often impaired earlier than LV GLS and before structural changes, such as increased ventricular wall thickness, become apparent. Not including this variable may compromise the model's accuracy and sensitivity for the early detection of cardiac amyloidosis. Another important point is that the study included a great majority of patients with a confirmed diagnosis of CA, which restricts the phenotypic variability of the sample and does not adequately reflect the real‐world clinical setting. This approach limits the model's ability to generalize and identify early or atypical cases. Ideally, training and external validation should be performed in more heterogeneous populations, including, for example, patients with multiple myeloma without cardiac involvement as well as individuals with LV hypertrophy of other etiologies, to enhance the robustness and clinical applicability of the tool. One last point to consider is that the study included only patients with pAL, and validation for patients with transthyretin amyloidosis, another frequent subtype of CA, is still needed. The prognostic value of the proposed nomogram is yet to be evaluated.

The proposed nomogram is a welcome and valuable contribution to the field, and the work of Lin et al. [7] represents an important step toward advancing echocardiographic assessment of CA. When properly tested and validated in diverse clinical populations, this approach has the potential to become a powerful additional tool for the early and accurate detection of the disease. Such capability is particularly relevant in the current therapeutic landscape, where timely diagnosis can enable the initiation of specific treatments that may alter the natural history of both AL and ATTR amyloidosis [11, 12].

## Conflicts of Interest

The authors declare no conflicts of interest.

## Ethics Statement

The authors have nothing to report.

## Consent

The authors have nothing to report.

## Data Availability

The authors have nothing to report.

## References

[1] A Martinez‐Naharro , AJ Baksi , PN Hawkins , et al., “Diagnostic Imaging of Cardiac Amyloidosis,” Nature Reviews Cardiology 17 (2020): 413–426. 32042139 10.1038/s41569-020-0334-7

[2] Y Tian and H Liu , “Advances and Challenges in Echocardiographic Diagnosis and Management of Cardiac Amyloidosis,” International Journal of Cardiovascular Imaging 41 (2025): 1021–1037. 40009119 10.1007/s10554-025-03362-5

[3] LG Gilstrap , F Dominici , Y Wang , et al., “Epidemiology of Cardiac Amyloidosis‐Associated Heart Failure Hospitalizations among Fee‐for‐Service Medicare Beneficiaries in the United States,” Circulation. Heart failure 12 (2019): e005407. 31170802 10.1161/CIRCHEARTFAILURE.118.005407 PMC6557425

[4] MW Bloom and PD Gorevic , “Cardiac Amyloidosis,” Annals of Internal Medicine 176 (2023): ITC33–ITC48. 36913688 10.7326/AITC202303210

[5] TA McDonagh , M Metra , M Adamo , et al., “2021 ESC Guidelines for the Diagnosis and Treatment of Acute and Chronic Heart Failure,” European Heart Journal 42 (2021): 3599–3726. 34447992 10.1093/eurheartj/ehab368

[6] SAM Cuddy , M Chetrit , M Jankowski , et al., “Practical Points for Echocardiography in Cardiac Amyloidosis,” Journal of the American Society of Echocardiography 35 (2022): A31–A40. 36064258 10.1016/j.echo.2022.06.006

[7] – Q Lin , X Li , Z Tan , et al., “Echocardiographic Nomogram Model: A Tool for Assessing Cardiac Involvement in Patients With Systemic Amyloidosis,” Echocardiogr 42 (2025): e70252. 10.1111/echo.70252 40782094

[8] ED Pagourelias , J Duchenne , O Mirea , et al., “The Relation of Ejection Fraction and Global Longitudinal Strain in Amyloidosis: Implications for Differential Diagnosis,” JACC Cardiovasc Imaging 9 (2016): 1358–1359. 26897665 10.1016/j.jcmg.2015.11.013

[9] XH Liu , JY Shi , DD Zhang , et al., “Prognostic Value of Left Atrial Mechanics in Cardiac Light‐Chain Amyloidosis With Preserved Ejection Fraction: A Cohort Study,” BMC Cardiovascular Disorders 22 (2022): 175. 35428181 10.1186/s12872-022-02589-7 PMC9013068

[10] A Aimo , I Fabiani , A Giannoni , et al., “Multi‐Chamber Speckle Tracking Imaging and Diagnostic Value of Left Atrial Strain in Cardiac Amyloidosis,” European Heart Journal ‐ Cardiovascular Imaging 24 (2023): 130–142. 10.1093/ehjci/jeac057 35292807

[11] E Kastritis , G Palladini , MC Minnema , et al.; ANDROMEDA Trial Investigators , “Daratumumab‐Based Treatment for Immunoglobulin Light‐Chain Amyloidosis,” New England Journal of Medicine 385, no. 1 (2021): 46–58. 34192431 10.1056/NEJMoa2028631

[12] CT Mesquita , P Schwartzmann , EB Correia , et al., “Patisiran Treatment in the Brazilian Subpopulation of the Phase 3 APOLLO‐B Study in Transthyretin Amyloidosis With Cardiomyopathy: Post Hoc Analysis,” Arquivos Brasileiros De Cardiologia 122, no. 4 (2025): e20240568. 40243852 10.36660/abc.2024568 PMC12107767

